# Effect of Ball Mill Treatment on the Physicochemical Properties and Digestibility of Protein Extracts Generated from Scallops (*Chlamys farreri*)

**DOI:** 10.3390/ijms19020531

**Published:** 2018-02-09

**Authors:** Di Wu, Chao Wu, Hui Chen, Zhenyu Wang, Cuiping Yu, Ming Du

**Affiliations:** School of Food Science and Technology, National Engineering Research Center of Seafood, Dalian Polytechnic University, Dalian 116034, China; m13039998695@163.com (D.W.); wuchaojiangnan@126.com (C.W.); realcrital@126.com (H.C.); wangzhenyu@dlpu.edu.cn (Z.W.); yuguiyan@126.com (C.Y.)

**Keywords:** *Chlamys farreri*, protein, ball mill treatment, surface hydrophobicity, foaming properties, digestibility

## Abstract

The effects of ball mill treatment (0, 2, 4, 6, 8, and 10 min) on the physicochemical and digestible properties of scallops (*Chlamys farreri*) protein (CFP) were investigated. The CFP particle size decreased with increasing ball-milling time. The content of free sulfhydryl (SH) of CFP increased from 13.08 ± 0.25 μmol/g protein to 18.85 ± 0.24 μmol/g protein when the ball-milling time increased from 0 min to 10 min. A sharp increase of the surface hydrophobicity index (H_0_) from 48.53 ± 0.27 to 239.59 ± 0.37 was found when the ball-milling time increased from 0 min to 4 min. Furthermore, the foaming capacity increased from 46.08 ± 6.12% to 65.11 ± 1.05% with increasing ball-milling time from 0 min to 6 min, after which it reached a plateau. SDS-PAGE results showed that ball mill treatment did not change the primary structure of CFP. Digestible properties of BMCFP simulated gastrointestinal digestion as a function of ball mill treatment were analyzed by Tricine-SDS-PAGE and nitrogen recovery index. After 60 min of simulated human gastro digestion, nitrogen recovery index of CFP had a significant rise from 42.01 ± 0.31% to 58.78 ± 3.37% as the ball-milling time increased from 0 min to 6 min. Peptides from hydrolysates of *Chlamys farreri* protein (CFP) were identified by ultraperformance liquidchromatographysystem coupled to a Synapt Mass Quadrupole Time-of-Flight Mass Spectrometer (UPLC-Q-TOF-MS). After 2 h and 4 h of simulated human duodenal digestion, the number of peptides with 7–10 amino acids length increased apparently with the ball-milling time increased. This study presents an approach to investigating the effect of the ball-milling process on the physicochemical and digestible properties of CFP, which may provide valuable information on the application of CFP as an ingredient in food products.

## 1. Introduction

Scallop (*Chlamys farreri*) is a kind of shellfish widely consumed and used as ingredients in the food industry. It is one of the major economic scallops cultured worldwide [1]. Different functional peptides derived from scallops had been extensively shown in the literature, such as the ability to protect against UV radiation [2], ACE inhibitor activity [2,3], and antibacterial properties [4,5,6]. Ding et al. [7] have studied the protective effect of polypeptide from *Chlamys farreri* against UV-irradiation on dermal fibroblasts. Han et al. [8] demonstrated that an octapeptide with molecular mass of 879 Da possessed antioxidant effects. In the food industry, Hayes et al. [9] reported that proteins and peptides extracted from marine sources could be used as stabilizing and thickening agents, protein replacements, and gelling agents. Protein and peptides can also have beneficial properties for human health and have demonstrated activities including anticoagulant, antioxidant, antihypertensive, and antibacterial activities. Garcia et al. [10] reported that fish feeding represented over 50% of the operating costs in intensive aquaculture, with protein being the highest dietary requirement of shellfish. However, few studies were carried out on scallop protein, which might be a potential dietary protein resource.

Superfine grinding, which is widely applied to reduce particle size and optimize reactive surface, has shown a great potential in producing proteins with suitable sensory and physicochemical properties [11,12]. Studies showed that superfine grinded powders exhibited higher protein solubility values [13], water holding capacities [14], and smaller particle size [15]. Among superfine grinding technologies, ball-milling is a high-efficiency and green processes to reduce particle size in the food industry. Several researchers used ball-milling to modify the characteristics of food materials, including mushrooms power [15], soy protein isolate [16], and whey protein concentrates [17]. According to the study by Liu et al. [16], the ball mill treatment increased the solubility and water-holding capacities of the micronized peels. Ball-milling provided a mechanochemical effect through combined friction, collision, and shear resulting from the grinding balls and the container wall by repeated high energy impact [18]. Ball-milled powders are easier to incorporate into food structure than those obtained through traditional grinding methods. After ball-milling, the solubility of nutritive components increases, and these components can be easily absorbed by the human body [19]. However, few studies focused on the effects of ball-milling on seafood proteins. Marine bioactive peptides and small proteins may also have applications as nutraceuticals, pharmaceuticals, and cosmeceutical ingredients [10]. Thus, scallops proteins extracted were used as model seafood proteins in the present study.

In order to investigate the effect of ball mill treatment on the physicochemical properties of CFP, particle size, protein composition, sulfhydryl group contents, surface hydrophobicity index, and foaming properties were investigated. The digestibility of the ball-milled CFP were studied by analyzing nitrogen recovery index and protein composition of samples at different digesting stages. He et al. [20] suggested marine proteins might become important protein resources for the generation of novel bioactive peptides that might result following enzymatic hydrolysis. The peptides in hydrolysates were identified by UPLC-Q-TOF-MS. Based on that, the relationship between ball-milling time and physicochemical properties or digestibility was discussed.

## 2. Results and Discussion

### 2.1. Effect of Ball-Milling of Protein Composition and Microstructure of CFP

**Figure 1:**
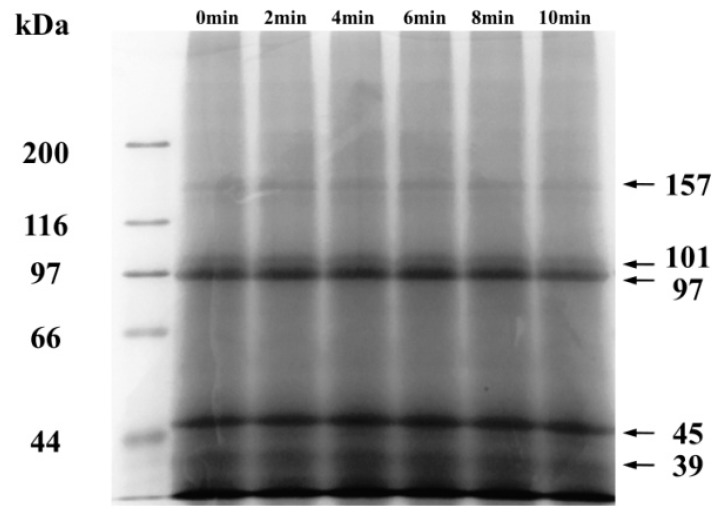
Reducing sodium dodecyl sulfate-polyacrylamide gel electrophoresis (SDS-PAGE) of ball-milled scallops (*Chlamys farreri*) protein (CFP) (0, 2, 4, 6, 8, and 10 min). The molecular weights of protein marker were 200 kDa, 116 kDa, 97 kDa, 66 kDa, and 44 kDa.

The effects of ball mill treatment on the primary structure of CFP were analyzed by reducing SDS-PAGE (Figure 1). The electrophoresis bands are distributing at 157 kDa, 101 kDa, 97 kDa, 45 kDa, and 39 kDa. Benedé et al. [21] reported that CFP contained actin and tropomyosin, which were distributing at 44.3 kDa and 35 kDa, respectively. Lyu et al. [22] showed *Chlamys farreri* had characteristics of electrophoresis bands at 97 kDa and 36 kDa. As shown in Figure 1, the protein distribution of CFP was not affected by ball milling. Liu et al. [16] came to the same results that no changes of the primary structure of soybean protein isolate were found after ball mill treatment.

**Figure 2:**
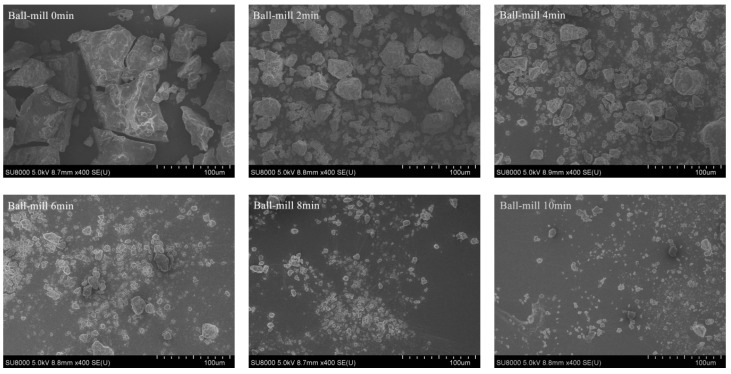
Effects of ball-milling time (0, 2, 4, 6, 8, and 10 min) on the microstructure of CFP.

SEM was used to investigate the effect of ball-milling on the microstructure of CFP. Figure 2 shows a set of SEM images of BMCFP at a magnification factor of 400-fold.The samples (Figure 2) obtained after ball mill treatment were of regular appearance with smaller size than the untreated one (Figure 2 0 min). As the ball-milling time increasing, more and more small particles appeared in the field of vision, which was in agreement with the results of Liu et al. [16]. Zhao et al. [18] reported that the particle size of powders decreased significantly after ball mill treatment observed by scanning electron microscopy. The longer the time of ball-milling took, the smaller the size of CFP particles. Moreover, as the ball-milling time increasing, particles were predominantly consisted of broken granules with a fragmented surface, which was presumably caused by the impact, shear, and friction effect of ball-milling [23]. After ball-milling for 8 min, the medium diameter was about 1–2 μm. With a prolonged ball-milling time (10 min), no apparent further variation of the granule morphology was observed. Li et al. [24] reported that the shape of the particles after ball milling was spherical, whereas the non-ball-milled particles were irregular, and he found that high collision and friction of ball-milling change the starch structural features, e.g., granule morphology, crystallinity, and molecular weight.

### 2.2. Effect of Ball-Milling on Sulfhydryl (SH) Groups

**Figure 3:**
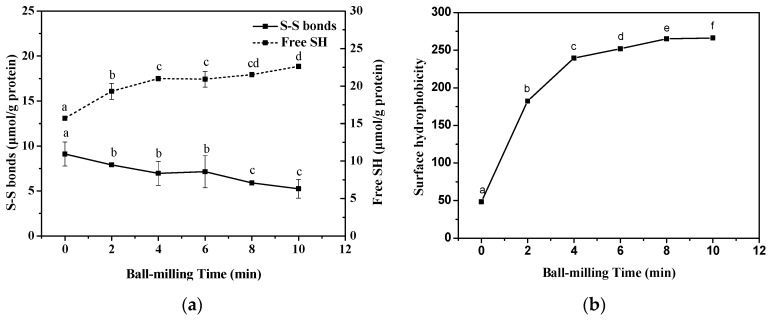
Effects of ball mill treatment (0, 2, 4, 6, 8, and 10 min) on the free sulfhydryl (SH) and disulfide (S-S) bonds (**a**) and surface hydrophobicity (**b**). Different lower case letters denote significantly differences (*p <* 0.05).

Sulfhydryl (SH) groups and disulfide (S-S) played important roles in the formation of secondary and tertiary structures of proteins [16,25]. Figure 3a shows the effect of ball-milling on the free SH-group and S-S bonds of CFP. With the increasing of ball-milling time, the free SH groups increased from 13.08 ± 0.25 μmol/g protein to 18.85 ± 0.24 μmol/g protein. Meanwhile, the S-S contents of CFP decreased from 9.11 ± 1.33 μmol/g protein to 5.24 ± 1.03 μmol/g protein. Sun et al. [17] also observed that ball mill treatment reduced the free SH groups of whey protein concentrate. After ball-milling, the buried SH groups got exposed, which could contact with oxygen in the air and mechano-chemistry generated hydrogen peroxide forming S-S bonds [26].

### 2.3. Effect of Ball-Milling on Surface Hydrophobicity (H_0_) Index

Hydrophobic interactions between the protein molecules can induce random aggregation, which could change the intermolecular interaction between protein and protein or protein and lipid. In Figure 3b, as the ball-milling time increasing, H_0_ of BMCFP has a sharp rise from 48.53 ± 0.27 to 239.59 ± 0.37 with increasing ball-milling time from 0 min to 4 min and small increase from 251.97 ± 0.24 to 266.26 ± 0.23 when ball-milling time was increased from 6 to 10 min. These indicated that more hydrophobic sites buried within the protein became exposed. Sun et al. [17] also reported that the H_0_ of whey protein concentrate increased from 99.8 to 175.7 after ball mill treatment. Liu et al. [16] investigated the effect of ball-milling on soybean protein isolate and found that H_0_ of SPI increased steady from 2058 ± 85 to 5051 ± 54 with increasing ball-milling time from 0 to 10 min.

### 2.4. Effect of Ball-Milling on Foaming Capacity and Foaming Stability

**Figure 4:**
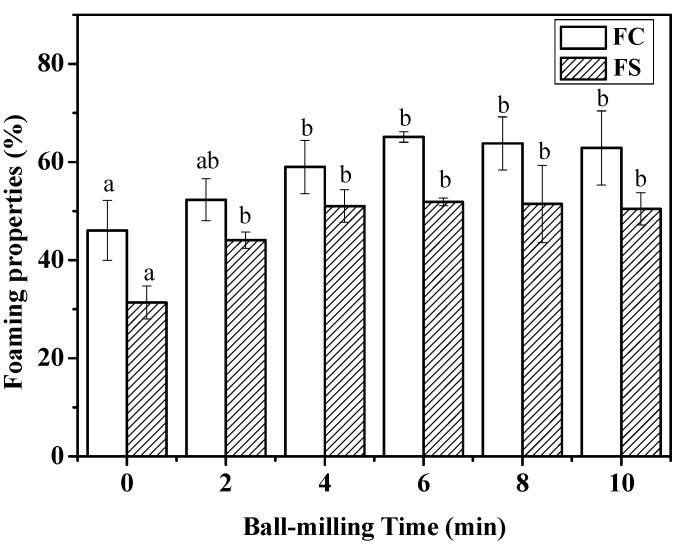
Effects of ball-milling time (0, 2, 4, 6, 8, and 10 min) on foaming capacity (FC) and foaming stability (FS) of CFP. Different lowercase letters denote significantly different (*p* < 0.05) from each other. Error bars show the variations of three determinations in terms of standard error of mean.

Foam can be defined as a two-phase system consisting of air cells separated by a thin continuous liquid layer [27]. Proteins, as a kind of amphiphilic substance, are ideally more suited than small molecular weight surfactants to act as macromolecular surfactants in foam-type products [28]. In addition to lowering the interfacial tension, proteins can form a continuous and highly viscous film at interfaces via complex intermolecular interactions. Figure 4 shows that foaming capacity (FC) was significantly (*p <* 0.05) increased from 46.08 ± 6.12% to 65.11 ± 1.05% with increasing ball-milling time from 0 min to 6 min. No significant changes of foaming capacity (FC) were found at longer treatment time. The same trend, as shown in Figure 4, also observed in the changes of foaming stability (FS) of CFP at different milling time. At the first 6 min of ball mill treatment, the foaming stability a significant (*p* < 0.05) increased from 31.37 ± 3.39% to 51.03 ± 3.30%, and then it reached a plateau. The changes of foaming capacity and stability after ball mill treatment showed in accordance with the results of surface hydrophobicity index of CFP, which could be explained by that more hydrophobic sites buried in the interior of the CFP were exposed [29].

### 2.5. Effect of Ball-Milling on Digestibility during the Digestions

**Figure 5:**
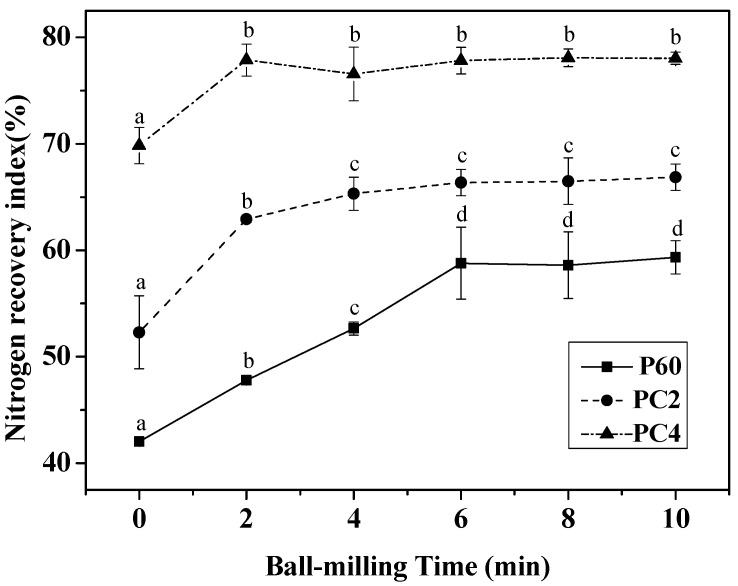
Effects of ball-milling time (0, 2, 4, 6, 8, and 10 min) on nitrogen recovery index simulated gastro duodenal digestion of CFP. Three curves, 60 min (P60) of gastric digests, 2 h (PC2), and 4 h (PC4) of gastro duodenal digests (60 min of gastric followed by 4 h of duodenal digestion), were showed digestibility of BMCFP during gastro duodenal digestion. *p* < 0.05.

The extent of nitrogen recovery index of BMCFP is shown in Figure 5. Three curves, 60 min (P60) of gastric digests, 2 h (PC2), and 4 h (PC4) of simulated in vitro gastrointestinal digestion (60 min of gastric followed by duodenal digestion), were shown digestibility of BMCFP during gastro duodenal digestion. As was observed, non-ball-milled CFP appeared to be of the lowest nitrogen recovery index. After 60 min simulated gastric digestion, a significant increase of nitrogen recovery index was found from 42.01 ± 0.31% to 58.78 ± 3.37% as the ball-milling time increasing from 0 to 6 min, and then it reached a plateau from 6 min to 10 min. Then, during 2 h and 4 h simulated duodenal digestion, it was observed that nitrogen recovery index of CFP within 4 min of ball-milling time increased significantly, while no further increase could be observed for the samples with a ball-milling time between 6 and 10 min. At the end of simulated in vitro gastrointestinal digestion, no significant difference of nitrogen recovery index was shown in different BMCFP. 

**Figure 6:**
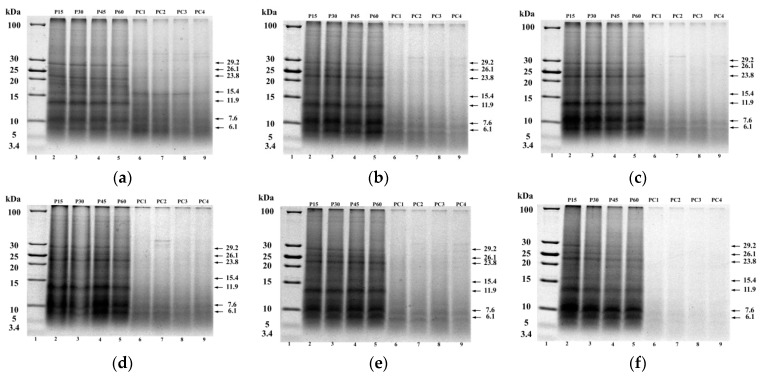
Tricine-SDS-PAGE of ball-milled CFP at different digesting time. (**a**–**f**), Tricine-SDS-PAGE image of CFP (0, 2, 4, 6, 8, and 10 min) after in vitro gastric and gastro duodenal digestions with simulated. Lane 1: molecular mass marker, lane 2–5: 15 min (P15), 30 min (P30), 45 min (P45), 60 min (P60) of gastric digests; lane 6–9: 1 h (PC1), 2 h (PC2), 3 h (PC3), 4 h (PC4) of gastro duodenal digests (60 min of gastric followed by 4 h of duodenal digestion).

Figure 6 shows protein composition of BMCFP samples collected at different digesting time. Seven bands with molecular masses of 29.2, 26.1, 23.8, 15.4, 11.9, 7.6, and 6.1 kDa were observed in the samples digested by pepsin for 15 min. Furthermore, the intensity of the bands with molecular weight of 7.6 and 6.1 kDa increased with increasing ball-milling time from 0 to 10 min. For all the samples, the intensity of these seven bands decreased when digesting time was increased from 15 min to 60 min, and an apparent decrease could be observed for the samples with a 10 min ball mill treatment. And the intensity of the bands with molecular weight of 29.2, 26.1, and 23.8 kDa decreased with increasing digestion time. When subjected to digesting by pancreatin, the protein bands disappeared even at 1 h of digesting time. Stefania et al. [30] also observed that the process of pancreatic hydrolysis of ovalbumin was rapid. The intensities of the protein bands obviously decreased with increasing ball-milling time from 0 to 10 min.

### 2.6. Effect of Ball-Milling on the Number of Identified Peptides

MS analysis was applied to investigate the effect of different time (0–4 min) of ball-milling on the number and the length of peptides in the hydrolysates. As discussed above, apparent differences of nitrogen recovery index and protein composition could be found among samples with ball-milling time from 0 to 4 min. Thus, samples with these three ball-milling time were chose in this section to analyze the effect of ball mill treatment on the characteristics of peptides. 

As shown in Table 1, 60 min of gastric digests (P60), 2 h (PC2), and 4 h (PC4) of gastro duodenal digests (60 min of gastric followed by 4 h of duodenal digestion) were hydrolysis fragment name of different ball-milling time of CFP during gastro duodenal digestion. After searching database of *bivalve* in the Mascot, peptides were divided into four intervals (7–10, 11–15, 16–20, and 21–30 AA) based on the length of peptide. After 60 min simulated gastro digestion of CFP (P60), no differences could be found for peptides with 16–20 and 21–30 amino acids before and after ball mill treatment. However, for the fragment of 7–10 AA, the number of peptides increased to 33 after ball mill treatment. After 2 h (PC2) and 4 h (PC4) simulated duodenal digestion, with the ball-milling time increased, it was observed that the number of peptides gradually increased. Especially, the number of peptide with 7–10 amino acids length increased from 80 to 120 and 354 to 392 for PC2 and PC4, respectively. After being simulated duodenal digestion for 4 h by pancreatin, the fragment of 21–30 AA no longer existed for the samples with 2 and 4 min ball mill treatment. Moreover, increases of the number of peptides with 16–20 and 7–10 amino acids were found when digesting time was increased from 2 h to 4 h. Overall, as ball-milling time increased, the number of peptides with 7–10 amino acids increased apparently in the hydrolysates of different digesting time.

## 3. Materials and Methods

### 3.1. Materials

*Chlamys farreri* were purchased from a local market (Dalian, Liaoning, China). Ethylene diamine tetraaceticaciddisodiumsalt (Na_2_EDTA), sodium dihydrogen phosphate, sodium hydrogen phosphate, DTNB, bromophenol blue sodium salt (BPB), β-mercaptoethanol, acrylamide, N, N′-Methylene-bis (acrylamide), Tricine, G-250 were purchased from Sigma Chemical Co. (St. Louis, MO, USA). All other chemicals and reagents used in this study were analytical and HPLC grade.

### 3.2. Generation of CFP Extract

The *Chlamys farreri* was homogenated with a high-speed homogenizer (T25, IKA, Guangzhou, China). Then, it was dissolved with deionized water in a 1:4 ratio (*w*/*v*). pH was set up to 11.0 by adding 1 M NaOH. The mixture were heated for 40 min in a 50 °C water bath. Then, the mixture was centrifuged at 10,000*× g* for 20 min at 20 °C (CR 412, Jouan, Saint-xHerblain, France). The protein concentration of supernatant was measured by Kjeldahl method [31]. Then, the supernatant was lyophilized and stored in a dryer for subsequent investigation.

### 3.3. Ball Mill Treatment of CFP

A mixer BM equipment (Mixer Mill MM400, Retsch Technology, Haan, Germany) was applied to produce ball-milled CFP (BMCFP) powder. Two stainless steel balls (25 mm φ) were put into two grinding jars (50 mL), respectively. SPI samples (3 g) were weighed accurately and added to each grinding jar [18]. The memory mode was set at P8. The parameter value of frequency was set as 20 Hz. The ball-milling time was set as 0, 2, 4, 6, 8, and 10 min to obtain BMCFP powder.

### 3.4.Sodium Dodecyl Sulfate-Polyacrylamide Gel Electrophoresis

Sodium dodecyl sulfate-polyacrylamide gel electrophoresis (SDS-PAGE) of BMCFP followed the method of Laemmli et al. [32] (1970) using 3% stacking gel and 12.5% separating gel. Analysis of CFP hydrolysates by Tricine-SDS-PAGE was conducted according to the method by Schägger et al. [33]. The concentrations of stacking and separating gels were 4% and 16%, respectively. The samples were adjusted to the same protein concentration and mixed with dissolving buffer (4% SDS, 20% glycerol, 0.125 M Tris-HCl buffer, and 0.01% bromophenol blue, pH 6.8). Each of the samples was mixed with β-mercaptoethanol 0.02% (*w*/*w*) and heated for 5 min at 100 °C. 10 μL of each sample was loaded onto gels, and the electrophoresis was carried out at a constant current of 45 mA, 30 V for separating gel until all samples entered into the stacking gel, and 100 V for separation until end. After electrophoresis, the gel was fixed with a solution of 100 mM ammonium acetate dissolved in methyl alcohol/acetic acid (5/1, *v*/*v*) for 2 h. After fixing, gel was stained with 0.025% (*w*/*v*) Coomassie Blue G-250 in 10% (*v*/*v*) acetic acid for 2 h and destained by 10% (*v*/*v*) acetic acid. All images were analyzed by Quantity One software version 4.6.2.70 (Bio-Rad Laboratories, Hercules, CA, USA) based on the separation of MW standards.

### 3.5. Scanning Electron Microscopy (SEM)

The BMCFP samples were attached to double-sided adhesive tape. The morphological characteristics of the samples were evaluated using Scanning Electron Microscopy (PP3010T, Hitachi/Quorum, Tokyo, Japan). The samples were sprinkled on a double-sided sticky tape placed on aluminum stubs and covered with thin gold film (Ding et al. 2016).

### 3.6. Determination of Sulfhydryl (SH) Groups

The SH groups of BMCFP were determined according to the method of Hu et al. [26] with some modifications. BMCFP samples were dissolved in buffer A (0.086 M Tris, 0.09 M glycine, 4 mM Na_2_EDTA, pH 8.0). Traditionally, a pH of 8.0 was selected for detecting the SH content of protein when using DTNB. It was reacted with thiolate anion (S) for determination of total SH groups, then they were solubilised in buffer B (buffer A containing 6 M urea and 0.5% SDS) at a protein concentration of 2 mg/mL. The mixtures were incubated for 20 min in a 20 °C water bath and then centrifuged at 10,000× *g* for 15 min at 4 °C. The supernatant fractions were used for determine SH group content. Six hundred μL of supernatant was mixed with 4 mL of buffer and 400 μL of 20 mM DTNB (in the same buffer). The solutions were vortexed and allowed to stand for 15 min at room temperature, and absorbance was measured at 412 nm using a UV-VIS spectrophotometer. The supernatants in buffer without DTNB were used as blanks. A molar extinction coefficient of 1.36 × 10^4^ M^−^^1^ cm^−1^ was used for calculating micromoles of SH/gram of protein.

### 3.7. Determination of Protein Surface Hydrophobicity (H_0_) Index

Surface hydrophobicity of BMCFP was determined according to the method of Chelh et al. [34]. BMCFP (2 mg/mL) was dissolved in 20 mM phosphate buffer (pH 6.0). To 2 mL of protein solution, 400 μL of 1 mg/mL BPB (in distilled water) was added and mixed well. Two mL 20 mM phosphate buffer (pH 6.0) and 1 mg/mL BPB (in distilled water) were used as a control. Samples and control were kept for 10 min at room temperature. Then, they were centrifuged at 4000× *g* for 15 min at room temperature. The absorbance of the supernatant (diluted 1/10) was measured at 595 nm against a blank of phosphate buffer. The amount of BPB bound is given by the formula

(1) $$\mathrm{BPB}\mathrm{bound}(\mu g)=\frac{400\;\mu g\;\times \;\left(A_{\mathrm{control}}\;-{\;A}_{\mathrm{sample}}\right)}{A_{\mathrm{control}}}$$

where A_sample_ is the sample absorbance at 595 nm and A_control_ is the control absorbance at 595 nm.

### 3.8. Foaming Properties

The foaming capacity (FC) and the foaming stability (FS) were determined according to the method of Makri et al. [28] and Garcia et al. [35]. The foams were prepared by air dispersion, with an Ultra-Turrax (IKA) T25 high-speed homogenizer at 4000 rpm for 5 min, in 25 mL of CFP solution (pH 8.0), which contained 0.2% (*w*/*v*) protein. The foams were then poured in a volumetric cylinder, and the initial foam volume along with the foam volume after 30 min were measured. For the evaluation of foam capacity (FC), foam volume at time 0 min was regarded and for the evaluation of foam stability (FS) the aqueous phase volume at time 30 min was regarded. These measurements took place in triplicate, and the values given were the mean values of the three measurements.

(2) $$\mathrm{FC}\%=\frac{V_{\mathrm{foam}\;0}}{V_{\mathrm{liquid}}}\;\times \;100$$

(3) $$\mathrm{FS}\%=\frac{V_{\mathrm{foam}\;30}}{V_{\mathrm{liquid}}}\;\times \;100$$

where V_liquid_ is the initial volume of protein solution before homogenization (mL), V_foam 0_ is the initial volume of foam generated after homogenization (mL), and V_foam 30_ is the volume of foam generated after 30 min homogenization (mL) at time 30 min.

### 3.9. Digestibility Determination

The in vitro gastrointestinal digestion of BMCFP was simulated using pepsin and pancreatin according to the method of Schmelzer et al. [36], with some modifications. One gram of CFP of different ball-mill time was mixed with 100 mL of distilled water, and human chewing was simulated using stomacher. The pH was adjusted to 2.0 with 0.2 M HCl at 37 °C, and the stomach phase was simulated by adding pepsin (Sigma-Aldrich P7125, Saint Louis, MI, USA) at a 3:100 (E:S) ratio. After digesting for 1 h, the enzyme was inactivated by adjusting the pH to 7.0 with 0.2 M NaOH. Then, pancreatin (Sigma-Aldrich P3292, Milan, Italy) was added at a ratio of 3:100 (E:S) to simulated intestinal phase. After 4 h of digestion at 37 °C, enzyme activity was terminated by heating for 5 min at 100 °C. During the digestion, the sample of 15 min, 30 min, 45 min, and 60 min of pepsin hydrolysis and 1 h, 2 h, 3 h, 4 h of pancreatic hydrolysis were collected for analysis. The reaction mixture was centrifuged at 10,000× *g* for 20 min at 4 °C to remove large particles, the supernatant was frozen and kept at −30 °C until analysis. 

The reactions were stopped by heating for 5 min at 100 °C, and the protein precipitates were removed by centrifugation at 10,000× *g* for 20 min at 4 °C. The soluble nitrogen in the supernatants was determined by Kjeldahl method. The N release during the digestion was calculated as

(4) $$N\mathrm{release}\%=\frac{N_{s}{\;-\;N}_{0}}{N_{\mathrm{tot}}}\times 100$$

where N_s_ (mg) issoluble nitrogen in supernatant phase, N_0_ (mg) soluble nitrogen at 0 min, and N_tot_ (mg) is total nitrogen of protein [22].

### 3.10. UPLC-Q-TOF Analysis of Hydrolysate

A Cleanert S C18-SPE column (Agela Technologies Inc., Wilmington, DE, USA) was used to desalt hydrolysate obtained by the in vitro gastrointestinal digestion as described by Burton et al. [37], with some modifications. The desalted samples were then subjected to analysis on a UPLC-Q-TOF system coupled to a Synapt Mass Quadrupole Time of Flight Mass Spectrometer (Bruker Daltonik, Bremen, Germany). Samples (25 μL) were loaded onto a Thermo Scientific ultra high-performance liquid chromatography (UPLC) instrument and separated using a mobile phase composed of formic acid in water (0.1% *w*:*v*) and acetonitrile in formic acid (0.1% *w*:*v*). The hydrolysis were then loaded onto a 4.6 mm × 150 mm C18 column (Phenomenex, Torrance, CA, USA) with a particle size of 3.0 μm. MS/MS spectra of the top 10 intense ions in the MS scan were acquired in automated data-dependent acquisition mode. Peptide sequences were determined by the Mascot searching program on the bivalve database download from NCBI based on the MS/MS spectra [38]. Automated spectral processing, peak list generation, and database search for the identification of the peptides were performed using Mascot v1.4.0.38 software with analyzing on peaks in the *m*/*z* range of 50–2200 and the intensity threshold (TIC AllMSn) pos. was 1000. The maximum number of compounds was set as 10 million. Mass tolerance was 10 ppm for MS and 0.03 Da for MS/MS ions and allowed for up to one missed proteolytic sites with no trypsin for protein cleavage. The Swiss-Prot protein database was used to identify the peptides with an ions score threshold of 20, a significance threshold *p <* 0.05, FDR ≤ 1%. Each sample was analyzed in triplicate. All the MS data were the combination of the peptides from three separate experiments

### 3.11. Statistical Analysis

In this study, all measurements were carried out in triplicate. Data was performed using the SPSS 16.0 software (SPSS Inc., Chicago, IL, USA), and the data were subjected to the analysis of variance (ANOVA). The figures illustrated are one of the parallel experiments and the mean results were presented. Free SH, S-S bonds, and surface hydrophobicity with different ball-milling time were analyzed with one way ANOVA and post-hoc test HSD Tukey. The repeated measures general linear model was used to test differences in foaming capacity and foaming stability with the effects of ball-milling time, and the differences analyzed using post-hoc Tukey HSD tests. The digestibility of ball-milled CFP was contemplated in the model, and post-hoc Tukey HSD tests were used to check the differences. In all cases, the criterion for statistical significance was *p <* 0.05.

## 4. Conclusions

The present study demonstrated that ball mill treatment had apparent impacts on physicochemical properties and digestibility of scallop (*Chlamys farreri*) protein. A reduction of protein particle size was found with increasing ball-milling time. The foam capacity and foam stability of CFP increased when ball-milling time was increased from 0 to 6 min, which might be due to the increase of surface hydrophobicity after ball mill treatment. In hydrolysates of different stages of digesting, the number of peptides with 7–10 amino acids increased with increasing ball-milling time. Since the effect of ball mill treatment on physicochemical properties and digestibility of CFP were revealed, further studies are underway to investigate emulsion and gelling properties of BMCFP.

## Figures and Tables

**Table 1:** Effects of ball-milling time (0, 2, and 4 min) on the number and length of peptides from hydrolysis fragment after simulated gastro duodenal digestion of CFP.

Amino Acid Number of Peptides from Hydrolysis Fragment	7–10 AA	11–15 AA	16–20 AA	21–30 AA	Total Peptides
P60 0 min	24	10	1	1	36
P60 2 min	28	23	2	2	55
P60 4 min	33	16	2	1	52
PC2 0 min	80	21	6	1	108
PC2 2 min	120	38	5	1	164
PC2 4 min	138	45	4	1	188
PC4 0 min	354	109	12	2	477
PC4 2 min	385	87	13	0	485
PC4 4 min	392	85	14	0	491
